# Long-term functional and structural outcomes after large chorioretinectomy for ruptured globe following blunt trauma

**DOI:** 10.1186/s40942-023-00492-7

**Published:** 2023-08-31

**Authors:** Marco Mura, Danilo Iannetta, Marco Pellegrini, Leonore A Engelbrecht, Laura Sarti, Francesco Parmeggiani, Abdulrahman Badawi, Hassan Dhibi, Sulaiman Al Sulaiman

**Affiliations:** 1 Department of Translational Medicine, University of Ferrara, Ferrara, Italy; 2 Vitreoretinal Division, King Khaled Eye Specialist Hospital, Riyadh, Kingdom of Saudi Arabia; 3 Ophthalmology Unit, IRCCS Azienda Ospedaliero-Universitaria di Bologna, Bologna, Italy; 4 St. Anna University Hospital, Via A. Moro 8, 44124 Cona (Ferrara), Italy

**Keywords:** Vitrectomy, Chorioretinectomy, Globe rupture, Ocular trauma

## Abstract

**Background:**

The purpose of this study was to present a modified surgical technique involving pars plana vitrectomy with large chorioretinectomy for eyes with rupture of the globe due to severe ocular blunt trauma.

**Methods:**

This retrospective study included consecutive patients with rupture of the globe due to blunt trauma who were treated at the King Khaled Eye Specialist Hospital (Riyadh, Saudi Arabia). All patients underwent 25-gauge pars plana vitrectomy with large chorioretinectomies involving all the tissue around the posterior scleral wounds. Outcome measures included best-corrected visual acuity (BCVA), anatomical success and globe survival, rates of complications.

**Results:**

15 eyes of 15 patients were included. Mean BCVA was 2.88 ± 0.13 logMAR at presentation, and significantly improved to 0.83 ± 0.28 logMAR (P < 0.001), with 10 patients (67%) achieving a final BCVA ≥ 20/200. Anatomical success and globe survival were achieved in 11 (73%) and 15 (100%) of eyes, respectively. Postoperative complications included retinal detachment in 6 eyes (40%), epiretinal membrane in 6 (40%), hypotony in 4 (26%), PVR in 2 (13%).

**Conclusions:**

Pars plana vitrectomy with large chorioretinectomy is an effective treatment for globe rupture following severe blunt trauma, yielding good visual outcomes and anatomical success rates.

**Supplementary Information:**

The online version contains supplementary material available at 10.1186/s40942-023-00492-7.

## Background

Rupture of the globe is one of the most serious consequences of severe ocular blunt trauma, often resulting in permanent loss of vision [1–4]. The long-term prognosis is related not only to the initial injury sustained at the moment of trauma, but also to the subsequent wound-healing response and scar formation. In particular, proliferative vitreoretinopathy (PVR) occurs in 40–60% of eyes with open-globe injuries, leading to worse visual and anatomical outcomes. [5–7].

Several surgical strategies aiming at preventing or minimizing PVR after ocular trauma have been described. Zivojnovic first proposed a surgical technique to remove the incarcerated retina and scar tissue within a perforation site. [8] Subsequently, Kuhn et al. described the creation of a prophylactic chorioretinectomy to prevent PVR in eyes with perforating injuries. [9] In this technique, all retinal tissue incarcerated into the perforation site is removed using the vitrector, with diathermy applied to cauterize the retina and choroid in a 1-mm ring around the exit wound/impact site of a penetrating injury. [9].

In this study we present a modified surgical technique involving pars plana vitrectomy with large chorioretinectomy for rupture of the globe following severe ocular blunt trauma.

## Methods

This retrospective study included 15 eyes of 15 consecutive patients with rupture of the globe due to severe blunt trauma who were treated at the King Khaled Eye Specialist Hospital (Riyadh, Saudi Arabia) between January 2016 and March 2020. All patients except 1 were referred from other institutions after having a primary repair consisting of scleral wound closure using 7/0 or 8/0 vicryl sutures or 10/0 nylon sutures with restitution of globe integrity. The principles of the Declaration of Helsinki and good clinical practice guidelines were followed. The Institutional Review Board at the King Khaled Eye Specialist Hospital, Riyadh, Saudi Arabia declared that this type of retrospective study did not require IRB approval, in accordance with Arabic laws on human clinical trials.

All patients were classified as Type A (ruptured globe), grade 4 and 5, Zone II-III according to the Ocular Trauma Classification Group. [4] The following data were obtained from the patients’ charts: age, gender, etiology of the trauma, presence of vitreoretinal incarceration, presence and extent of detachment, presence and extent of choroidal detachment, time to primary surgical repair, type of tamponade agent, best-corrected visual acuity (BCVA) before and after surgery and anatomical outcome. Main outcome measurements included: BCVA, rates of PVR, anatomical success at the end of follow-up and globe survival. Anatomical success was defined as complete retinal attachment without silicone oil tamponade. Globe survival as complete retina attachment, normal (> 6 mmHg) intraocular pressure (IOP) and BCVA of light perception or better.

All surgical procedures were performed by an experienced surgeon (MM) under general anesthesia using the 25-gauge Alcon Constellation Total Pack (Alcon, Fort Worth, TX, USA) and BIOM III/IV viewing system (Oculus Optikgeräte GmbH, Wetzlar, Germany). When kissing choroidal detachment was present as assessed by B scan ultrasonography, choroidal drainage was first performed ab externo using one or multiple 3-mm full thickness radial beveled scleral incisions. This step was performed keeping the eyeball under high IOP of 60 mmHg via the 25-gauge infusion cannula placed in the anterior chamber. Once a satisfactory drainage of the suprachoroidal hemorrhage was achieved, two 25-gauge trocars were inserted perpendicularly to sclera 3 mm posterior to the limbus in the supero-temporal and supero-nasal quadrants. A chandelier light was placed 3 mm posterior to the limbus in the infero-nasal quadrant to facilitate bimanual surgical maneuvers. A pars plana lensectomy was performed in all phakic patients preserving the anterior capsule. The vitrectomy was performed as thoroughly as possible using triamcinolone acetonide to stain the vitreous cortex when not already made visible by the vitreous hemorrhage. When necessary, perfluorocarbon liquid (PFCL) was used to stabilize the retina at posterior pole. A large chorioretinectomy was performed in areas of vitreous and retinal incarceration, and in 6/15 cases, also in areas in which the suprachoroidal hemorrhage could not be completely drained. The extension of the chorioretinectomy was judged based on the presence of surrounding healthy tissue. Diathermy or endolaser retinopexy was used to delineate the area of chorioretinectomy. The chorioretinectomy was performed using the vitreous cutter until the bare sclera was visualized. To minimize active bleeding, the infusion pressure was elevated to 60 mmHg and a continuous barrage of laser photocoagulation was applied to the choriocapillaris and large choroidal vessels when visible. Very limited bleeding was observed during the procedure in cases with large scleral ruptures with severe disruption of the choroidal circulation or when large blood cloths were present at the site of the chorioretinectomy. After removal of damaged tissue and suprachoroidal hemorrhage, endolaser retinopexy was applied along the edge of the chorioretinectomy in healthy tissue to create a chorioretinal adhesion. Inferior peripheral iridectomy was performed in aphakic patients. PFCL was then exchanged for silicone oil directly, or after fluid-air exchange (Supplemental video 1). Since eyes with traumatic choroidal wounds are at risk for ocular venous air embolism, the correct positioning of the air infusion cannula was always confirmed before air-fluid exchange. [10].

Statistical analysis was performed using SPSS software for Windows version 25.0 (SPSS Inc, Chicago, Illinois, USA). Variables were reported as mean ± standard deviation, and parametric tests were used in the statistical analysis. BCVA was converted to LogMAR values, whereby counting fingers was converted to 1.40 logMAR, HM to 2.70 logMAR, light perception to 3.70 logMAR, absence of light perception to 4.70 logMAR.

## Results

Fifteen patients (12 males and 3 females) with a mean age of 35.0 ± 18.0 years (range 9–70 years) were included. Preoperative characteristics of eye injuries and clinical findings are shown in Table 1. The mechanism of injury was a blunt trauma in all patients. At presentation, mean BCVA was 2.88 ± 0.13 logMAR (range 2.60–3.0 logMAR).

**Table 1:** Preoperative characteristics of eyes with rupture of the globe due to blunt trauma

Postoperative complications	Eyes (%)
Lens injury	
Traumatic cataract	9 (60%)
Lens subluxation/dislocation	3 (20%)
Retinal injury	
Retinal detachment	11 (73%)
Retinal dialysis	2 (13%)
Vitreoretinal incarceration	12 (80%)
Suprachoroidal hemorrhage	9 (60%)
Proliferative vitreoretinopathy	6 (40%)

Patients underwent surgery with a mean interval time of 9.7 ± 4.3 days (range 0–15 days) from the primary repair. The mean number of surgeries excluding the primary repair was 2.5 ± 1.1 (range 1–4). The mean follow-up following surgery was 26.5 ± 11.3 months (range 12–42 months).

**Fig. 1:**
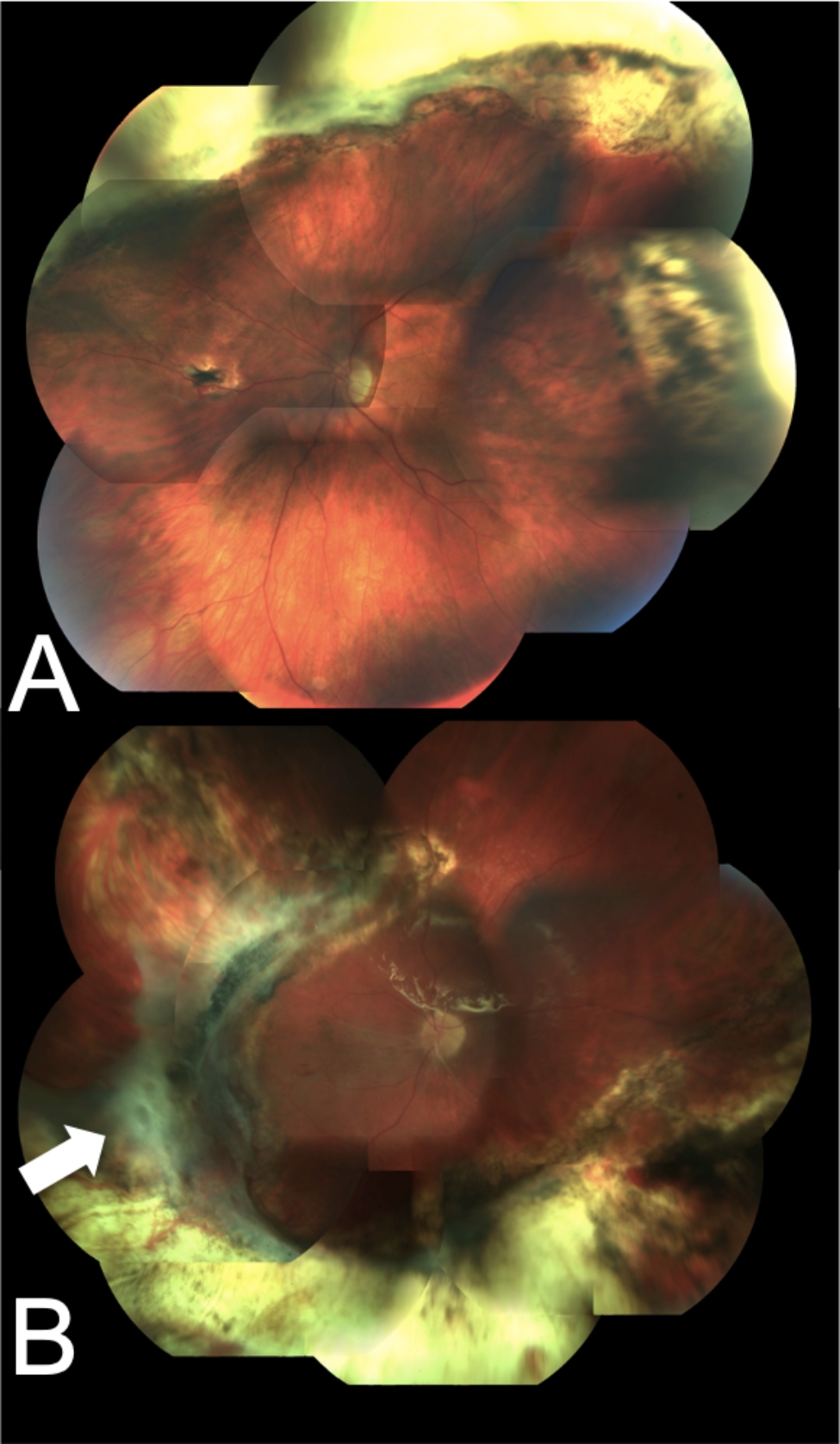
Posterior segment photographs of two representative patients who underwent pars plana vitrectomy with large chorioretinectomy for rupture of the globe due to blunt trauma. **A**, 12 months after surgery, the retina is attached and a large supero-temporal defect corresponding to the large chorioretinectomy is visible. BCVA was 0.6. **B**, 12 months after surgery, the retina is attached and a large infero-temporal defect corresponding to the large chorioretinectomy is visible, with an area of proliferative vitreoretinopathy at the edge of the chorioretinectomy (white arrow). BCVA was 0.2

Globe survival and final retinal attachment were achieved in all eyes (Fig. 1), while anatomical success occurred in 11/15 (73%), since 4 eyes were left under silicone oil due to persistent hypotony. The mean final BCVA was 0.83 ± 0.28 logMAR (1.40–0.60 logMAR), showing a significant improvement from baseline (P < 0.001). In total, 10 patients (67%) achieved a final BCVA ≥ 20/200. Postoperative complications included retinal detachment in 6 patients (40%), epiretinal membrane in 6 patients (40%), hypotony in 4 patients (26%), PVR in 2 patients (13%) (Table 2).

**Table 2:** Postoperative complications following pars plana vitrectomy with large chorioretinectomy for globe rupture due to blunt trauma

Postoperative complications	Eyes (%)
Retinal detachment	6 (40%)
Proliferative vitreoretinopathy	2 (13%)
Epiretinal membrane	6 (40%)
Endophthalmitis	0 (0%)
Hypotony	4 (26%)
Phtisis	0 (0%)

The total number of surgeries was higher in eyes with PVR at presentation compared to those without PVR (3.3 ± 0.8 vs. 2.0 ± 0.9; P = 0.017). Moreover, eyes with PVR at presentation showed worse final outcomes compared to those without PVR, with less eyes achieving a final BCVA ≥ 20/200 (33% vs. 89%) and lower anatomical success rate (50% vs. 89%).

## Discussion

Rupture of the globe is among the types of ocular injury with the poorest prognosis, representing an important public health problem that can result in lifelong sequelae with high psychosocial and economic costs. [11, 12] Large epidemiological studies estimated that no less than 60% of ruptured globes in the United States lead to legal blindness (as defined by a BCVA worse than 20/200) [13]. In most cases, poor clinical outcomes are associated with the occurrence of PVR, which usually develop at the scleral wound and ultimately leads to full-thickness retinal folds and tractional retinal detachment. [14] Therefore, novel surgical approaches aimed at preventing this complication are of great interest.

Prophylactic chorioretinectomies were initially proposed by Kuhn et al. as a treatment of perforating trauma associated with intraocular foreign bodies. [9] This procedure involves the complete removal of incarcerated vitreous and retinal tissue into the perforation site as well as surrounding exposed retinal pigment epithelium which may potentially proliferate and cause PVR. The zone of bare sclera around the impact site acts as a barrier between the scleral scar and the neighboring healthy retina. [9] While in eyes with perforating trauma the technique can be applied in a localized fashion around the exit wound, globe rupture is usually associated with large and irregular areas of scleral/choroidal/retinal defects. Thus, in this cohort of eyes with rupture of the globe we modified the surgical technique by performing large chorioretinectomies involving all the tissue around the posterior scleral wounds. In eyes with suprachoroidal hemorrhages, chorioretinectomies allowed the complete blood drainage with the advantage of preventing fibrous adhesions of the retinal tissue to the scleral wound. While there are other surgical indications in which a choroidectomy can be indicated, in ruptured globes the overlying retinal tissue is invariably compromised and a combined chorioretinectomy is therefore required.

Although ruptured globes are considered the most severe type of ocular trauma, [14, 15] the clinical outcomes in this study are similar to those reported in other series of perforating injuries treated with pars plana vitrectomy and prophylactic chorioretinectomy. [16]–[20] In particular, 67% of eyes from this series achieved a BCVA ≥ 20/200. This is in line with the results of Monteiro et al. (65% of eyes ≥ 20/200),^22^ and Weichel et al. (54% of eyes ≥ 20/200);^16^ while compares favorably with those of Ozdek et al. and and Kuhn et al. (only 31% and 18% of eyes ≥ 20/200, respectively). [17, 18].

With regards to PVR, the postoperative incidence observed in this study was 13%. Similar rates ranging from 6 to 20% were previously reported in other studies in which a chorioretinectomy was performed. [17]–[20] Conversely, up to 60% of eyes with severe ocular trauma develop PVR following pars plana vitrectomy alone. [21, 22] The low PVR rate in our series likely contributed to the good anatomical success of 73%, with final retinal attachment and globe survival achieved in all eyes. This confirms the efficacy of prophylactic chorioretinectomy in preventing PVR and the subsequent tractional retinal detachment. Thus, from the results of this study, large chorioretinectomies should be performed even for ruptured globes following blunt trauma with extensive posterior scleral wounds.

While the PVR rate was low, 40% of eyes developed epiretinal membranes which required peeling at the time of the silicone oil removal. In this respect, prophylactic internal limiting membrane peeling should be considered in patients undergoing such aggressive surgery to minimize epiretinal tissue growth.

The right timing in performing surgery following ocular trauma remains controversial. Kuhn advocated to perform pars plana vitrectomy as early as possible (ideally during the primary surgery when the wound is sutured, alternatively within 100 h from the injury). [18] Other authors suggested to wait a few days in order to minimize intraoperative bleeding and improve corneal clarity. [16], 23] In the present series, some cases were not referred for tertiary care until relatively late, when PVR had already developed. We noticed that patients who had PVR at presentation required more surgeries to reach retinal reattachment and had a worse final functional prognosis with higher rates of hypotony. This underlies the importance of prompt referral and early vitrectomy in order to prevent PVR formation and improve final BCVA and anatomical success rates.

Our study has several limitations including its retrospective design and the lack of a control group to evaluate the results of pars plana vitrectomy alone. This limits the generalizability of our findings. However, conducting a randomized controlled trial enrolling patients with severe ocular trauma would be problematic due to safety and ethical concerns.

## Conclusions

In conclusion, pars plana vitrectomy with large chorioretinectomy is an effective treatment for globe rupture following severe blunt trauma, yielding low rates of PVR. Early surgery might be important for anatomical success and final visual outcomes.

### Electronic supplementary material

Below is the link to the electronic supplementary material.

Supplemental video 1. Surgical technique of pars plana vitrectomy with large chorioretinectomy for globe rupture due to blunt trauma. A complete bimanual vitrectomy with scleral depression was performed with special attention to release any incarcerated vitreous and retina in the scleral rupture and remove completely the vitreous around this area. Perfluorocarbon liquid (PFCL) was injected to stabilize the posterior pole and the chorioretinectomy was performed using the vitreous cutter until the bare sclera was visualized. Subretinal fluid and PFCL was drained during under continuous air infusion. Endolaser was then applied around the chorioretiniectomy and air was exchanged for silicone oil

## Data Availability

The dataset used and analyzed during the current study is available from the corresponding author on reasonable request.

## List of abbreviations

PVR: proliferative vitreoretinopathy
BCVA: best-corrected visual acuity
IOP: intraocular pressure
PFCL: perfluorocarbon liquid
